# Theophylline-Loaded Pectin/Chitosan Hydrochloride Submicron Particles Prepared by Spray Drying with a Continuous Feeding Ultrasonic Atomizer

**DOI:** 10.3390/polym14214538

**Published:** 2022-10-26

**Authors:** Kuo-Chung Cheng, Chia-Chien Hu, Chih-Ying Li, Shih-Chi Li, Zhi-Wei Cai, Yang Wei, Trong-Ming Don

**Affiliations:** 1 Department of Chemical Engineering and Biotechnology, National Taipei University of Technology, Taipei 10608, Taiwan; 2 Department of Chemical and Materials Engineering, Tamkang University, New Taipei City 251301, Taiwan

**Keywords:** pectin, chitosan hydrochloride, submicron particles, ultrasonic atomizer, spray dryer, Ritger–Peppas model, control release

## Abstract

Pectin/chitosan hydrochloride (CHC) particles containing theophylline were prepared by a spray-drying apparatus coupled with a continuous feeding ultrasonic atomizer and a heating column. The formation of the submicron particles was investigated at various compositions of pectin solutions added with a chitosan hydrochloride or calcium chloride solution as a crosslinking agent. Scanning electron microscopic (SEM) images showed the pectin/chitosan hydrochloride particles had spherical and smooth surfaces. Depending on the feeding concentrations, the produced particles had diameters in the range of 300 to 800 nm with a narrow size distribution. Furthermore, the theophylline (TH)-loaded pectin/CHC particles were also prepared by the same apparatus. The TH release from the submicron particles in phosphate-buffered saline at 37 °C was monitored in real-time by a UV-Visible spectrophotometer. The Ritger–Peppas model could well describe the TH release profiles. All the diffusional exponents (*n*) of the release systems were greater than 0.7; thus, the transport mechanism was not a simple Fickian diffusion. Particularly, the *n* value was 1.14 for the TH-loaded particles at a pectin/CHC weight ratio of 5/2, which was very close to the zero-order drug delivery (*n* = 1). Therefore, the constant drug-release rate could be achieved by using the spray-dried pectin/CHC particles as the drug carrier.

## 1. Introduction

**Scheme 1:**
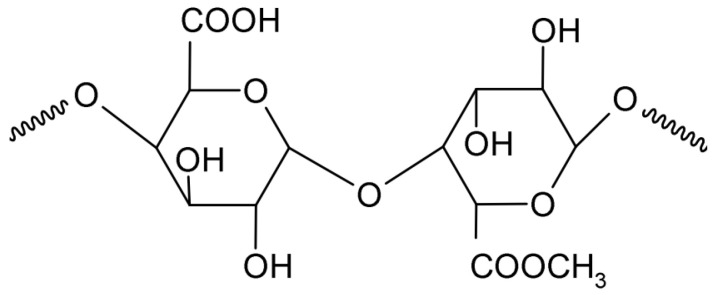
Pectin.

**Scheme 2:**
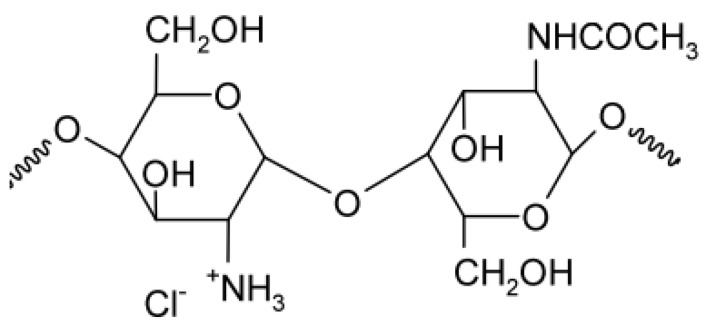
Chitosan hydrochloride.

Pectin is a natural anionic hetero-polysaccharide in many terrestrial plants’ cells (Scheme 1). It is mainly extracted from citrus fruit peel for applications in many food products, such as jellies, jams, and dietary fiber. Furthermore, pectin can be used in the pharmaceutical field owing to its good biocompatibility [1,2,3]. For example, pectin can be crosslinked with calcium ions, which is a suitable delivery system to escort drugs from the mouth to the colon [4,5]. On the other hand, chitosan and chitosan hydrochloride chitosan (Scheme 2) are cationic polysaccharide polymer derived from chitin, which can be found in the shells of shrimp, crabs, lobsters, etc. Chitosan can be used as a drug carrier because of its good mucoadhesive and anti-inflammatory properties. A polyelectrolyte complex of pectin and chitosan can be primarily formed by ionic interactions between the ionized carboxyl acid groups of pectin and amino groups of chitosan [6,7,8,9,10,11]. For example, vancomycin-loaded pectin/chitosan complex tablets had good mucoadhesive properties and a pH-dependent swelling sensitivity for colon delivery [8]. The anti-Alzheimer was encapsulated in chitosan/pectin microparticles of approximately 2 to 20 μm in diameter for nasal administration of tacrine hydrochloride [11].

**Scheme 3:**
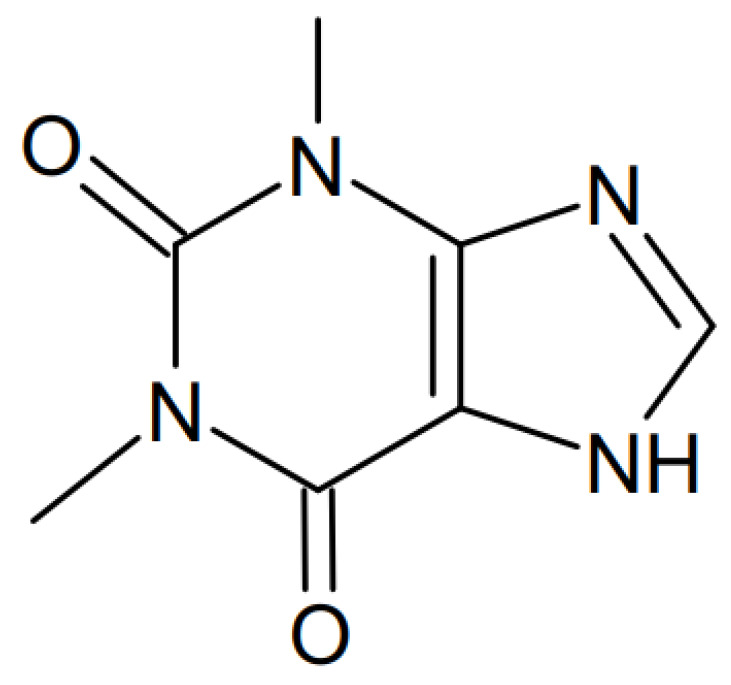
Theophylline.

Many submicron polymeric particles as drug carriers have exhibited excellent physicochemical properties and could overcome the biological barriers to delivering the loaded drug to the target site [12,13,14,15,16,17,18,19,20]. Resveratrol-loaded chitosan/pectin nanoparticles were prepared by using a complicated process including antisolvent precipitation and electrostatic deposition methods, and the complex particles could entrap resveratrol for a longer time [21].Theophylline (TH, Scheme 3) can be used for treating asthma and chronic obstructive pulmonary illnesses [22,23]. In the previous study, we demonstrated a simple spray-drying process to produce theophylline (TH)-loaded chitosan submicron particles [24]. The drops were produced by ultrasonic atomization from a mixture solution of chitosan, TH, and tripolyphosphate (TPP) in a conical flask, then dried in a heating column. However, as the initial precursor solution contained the crosslinking agent TPP, most chitosan was crosslinked with TPP before being atomized. Therefore, the crosslinking density of the spray-dried particles would be varied at different collecting times.

In this study, a modified spray drier coupled with two continuous feeding streams was set up. One stream contained a pectin solution, and the other one was filled with a crosslinker solution. They were then mixed and the formation of particles was investigated at various compositions of the pectin, chitosan hydrochloride, and theophylline (TH) in the aqueous solution. The new continuous feeding process could keep the concentration and composition of the precursor solution at a constant condition, and reduce the crosslinking reaction before spray drying. First, the pectin particles with the CHC were prepared at various concentrations of the feeding precursor solutions. The size and morphology of the spray-dried pectin/CHC particles were observed using a scanning electron microscope (SEM). The hydrodynamic diameters of the particles in the PBS were measured by using a dynamic light scattering (DLS) method. Theophylline (TH)-loaded pectin particles with a crosslinking agent, such as CHC or calcium chloride, were further fabricated by the twin-syringe, continuous-feeding ultrasonic atomizer. The TH release from the pectin/CHC particles in PBS was monitored using real-time UV-Visible spectroscopy. Finally, a release kinetic model was proposed to describe the drug release mechanism.

## 2. Materials and Methods

### 2.1. Materials

Pectin extracted from citrus with a molecular weight of approximately 20–40 kDa was purchased from Tokyo Chemical Industry (Tokyo, Japan). Chitosan hydrochloride (CHC) was obtained from Charming & Beauty Co., Ltd. (Taipei, Taiwan). Calcium chloride (CaCl_2_) was provided by Showa Chemical Industry (Tokyo, Japan). Theophylline (TH) was purchased from Acros Organics (Geel, Belgium), which was used as a model drug.

### 2.2. Preparation of Pectin/Chitosan Hydrochloride Microparticles

**Figure 1:**
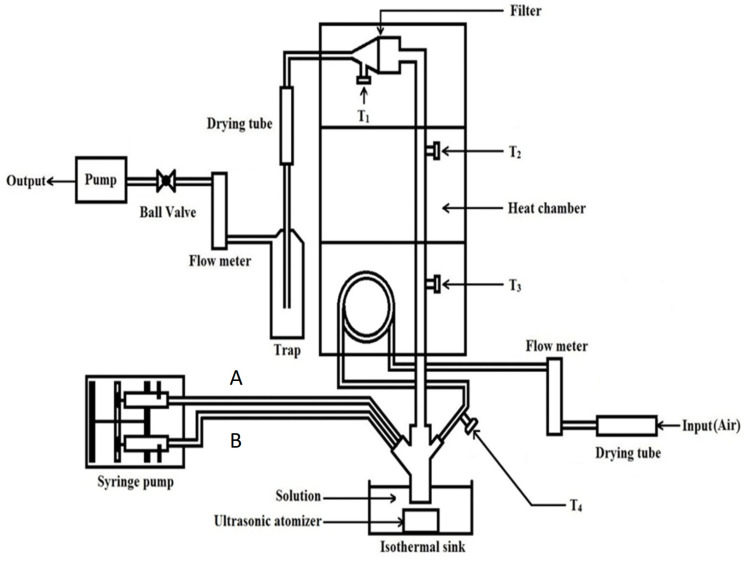
Spray drying apparatus coupled with a continuous-feeding ultrasonic atomizer, in which T_1_, T_2_ and T_3_ indicated the temperatures measured by thermocouples.

A homemade spray drier was set up using an ultrasonic atomizer with a continuous multiple feeding syringe pump and coupled with a heating column as shown in Figure 1. An aqueous pectin solution (0.05−0.15 mg/mL, coded as A) with or without theophylline (TH, 0.2 mg/mL) was filled into a syringe, and another aqueous solution containing CHC (0.1, 0.2 mg/mL) or calcium chloride (0.10 mg/mL) as a crosslinking agent (coded as B) was filled into the other syringe. These two solutions, A and B, were then fed into the atomizer cell at a constant flow rate of 0.125 mL/min by a syringe pump (NE-4000, NEW ERA Pump Systems Inc., Farmingdale, NY, USA). The spray drops of the mixed solution would float and be dried in a heated upstream airflow under 1.6 L/min. at controlled temperatures of T_1_ = 130 °C; T_2_ = 140 °C; and T_3_ = 150 °C. The dried particles were collected by a polytetrafluoroethylene membrane (MPT2247, 0.22 µm, ChromTech, Bad Camberg, Germany) and kept in a dry box for further study.

### 2.3. Characteristics of Pectin/Chitosan Hydrochloride Microparticles

For the morphology observation, the prepared particles were first sputter-coated with Au/Pd; then, the coated particles were observed by scanning electron microscopy (SEM, HITACHI S-3000H, Tokyo, Japan) at an accelerating voltage of 5 kV. Particle sizes and zeta potential of the spray-dried samples in phosphate-buffered saline (PBS, pH 6.8) at 37 °C were also analyzed by using the dynamic light scattering method (Nano Series, Malvern Panalytical Ltd., Almelo, Netherlands). Attenuated total reflection–Fourier transform infrared spectroscopy (ATR-FTIR, Spectrum 3, PerkinElmer, Waltham, MA, USA) was performed at room temperature in the range between 500–4000 cm^–1^ to detect the functional groups of the prepared particles.

### 2.4. The Release Profile of TH from the Prepared Pectin/Chitosan Hydrochloride Microparticles

5 mg of TH-loaded pectin/CHC microparticles was suspended in 0.5 mL of PBS (pH 6.8), and the solution was placed in a dialysis sack (Cellu.Sep T1, MWCO = 3500, Membrane Filtration Product, Inc., Seguin, TX, USA). The sack was further introduced into a release medium of 100 mL PBS, which was stirred at 100 rpm under 37 °C. The TH released into the buffer solution was measured by a UV-Visible spectrophotometer coupled with a dip probe (TP300-UV-VIS, Ocean Optics, Orlando, FL, USA) under real-time monitoring. The concentration of TH was determined by measuring the light absorbance at a wavelength of 272 nm with the help of a calibration curve constructed from the standard solutions of TH. By using that releasing test, the experimental drug loading contents (LC, %), and the encapsulation efficiency (EE, %) of the TH-loaded particles can be determined by the following equations:

(1) $$\mathrm{LC}(\%)=\;\frac{\mathrm{mass}\;\mathrm{of}\;\mathrm{drug}\;\mathrm{in}\;\mathrm{particles}}{\;\mathrm{mass}\;\mathrm{of}\;\mathrm{particles}}\times 100$$

(2) $$\mathrm{EE}(\%)=\;\frac{\mathrm{experimental}\;\mathrm{drug}\;\mathrm{loading}}{\;\mathrm{nominal}\;\mathrm{drug}\;\mathrm{loading}}\times 100$$

where the mass of the drug in the TH-loaded particles was estimated by the measured concentration of TH after releasing for three hours, and the nominal drug loading was calculated by the mass fraction of TH in the solid content of the fed precursor solution.

## 3. Results and Discussions

### 3.1. Characteristics of Pectin/Chitosan Hydrochloride Microparticles

**Figure 2:**
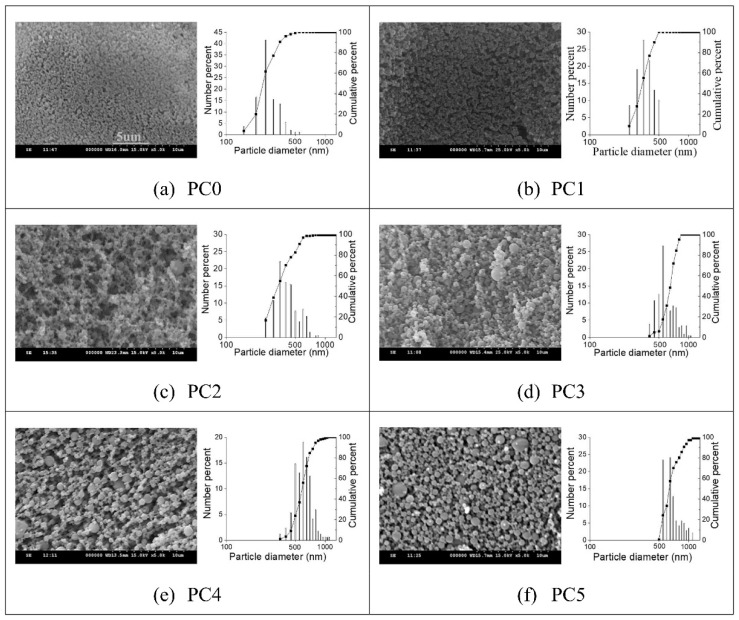
SEM images and corresponding particle size distributions of the pectin/CHC particles (PC0 to PC5) prepared from various solutions of pectin and CHC at different compositions.

**Figure 3:**
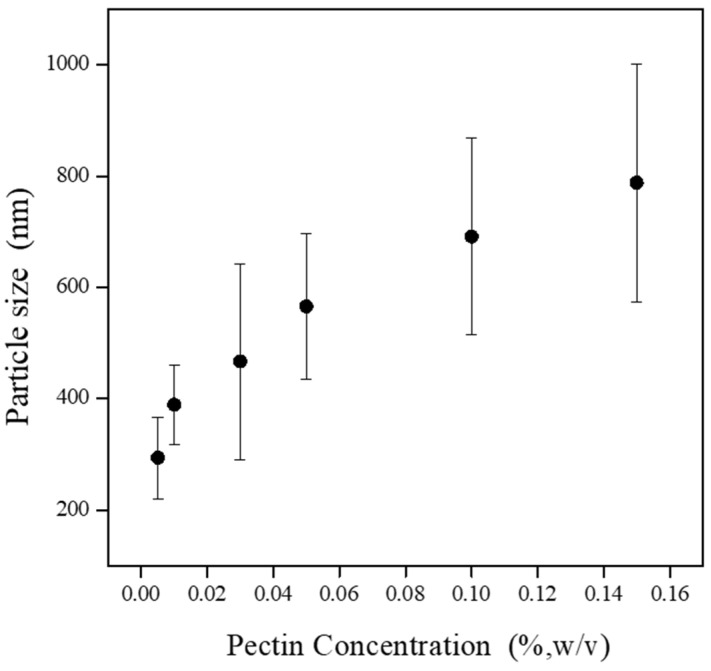
Variation in number average particle size (D_n_) of the spray-dried pectin/CHC particles with the pectin concentration, in which the weight ratio of pectin and CHC was kept at 5:1 as indicated in Table 1.

The morphology and particle size distribution of the spray-dried pectin/CHC complex particles prepared by the continuous-feeding solutions of the pectin with the CHC were observed by using the SEM as shown in Figure 2. Most of the pectin/CHC particles formed under drying at controlled temperatures: T_1_ = 130 °C; T_2_ = 140 °C; and T_3_ = 150 °C, had smooth surfaces and spherical shapes. Because the atomized drop contained higher solid content at a higher precursor concentration in the feeding solution, the size of the dried particles was expected to be larger once the water had been vaporized. The number average diameter size, D_n_, of the pectin/CHC particle calculated by the SEM thus increased with the increase in the precursor concentration as shown in Table 1 and Figure 3. For example, The D_n_ was approximately 294 nm for the particles made from the solutions of pectin and CHC at concentrations of 0.05 and 0.01 mg/mL (sample PC0), respectively. It was increased to 788 nm as the concentrations of pectin and CHC were increased to 1.5 and 0.3 mg/mL (PC5), respectively. The diversity of the particle size was usually described by the geometric standard deviation (G.S.D.) [24]. The G.S.D. value was 1.52 of the pectin/CHC made from the precursor solution with 0.3 mg/mL of the pectin and 0.06 mg/mL of the CHC (sample PC2). However, the values of the other samples were less than 1.4. It implies that the pectin/CHC submicron particles with a narrow particle size distribution can be prepared by using the continuous-feeding ultrasonic atomizer coupled with a heating column in the spray drier.

### 3.2. Characteristics of Theophylline-Loaded Pectin Particles

**Figure 4:**
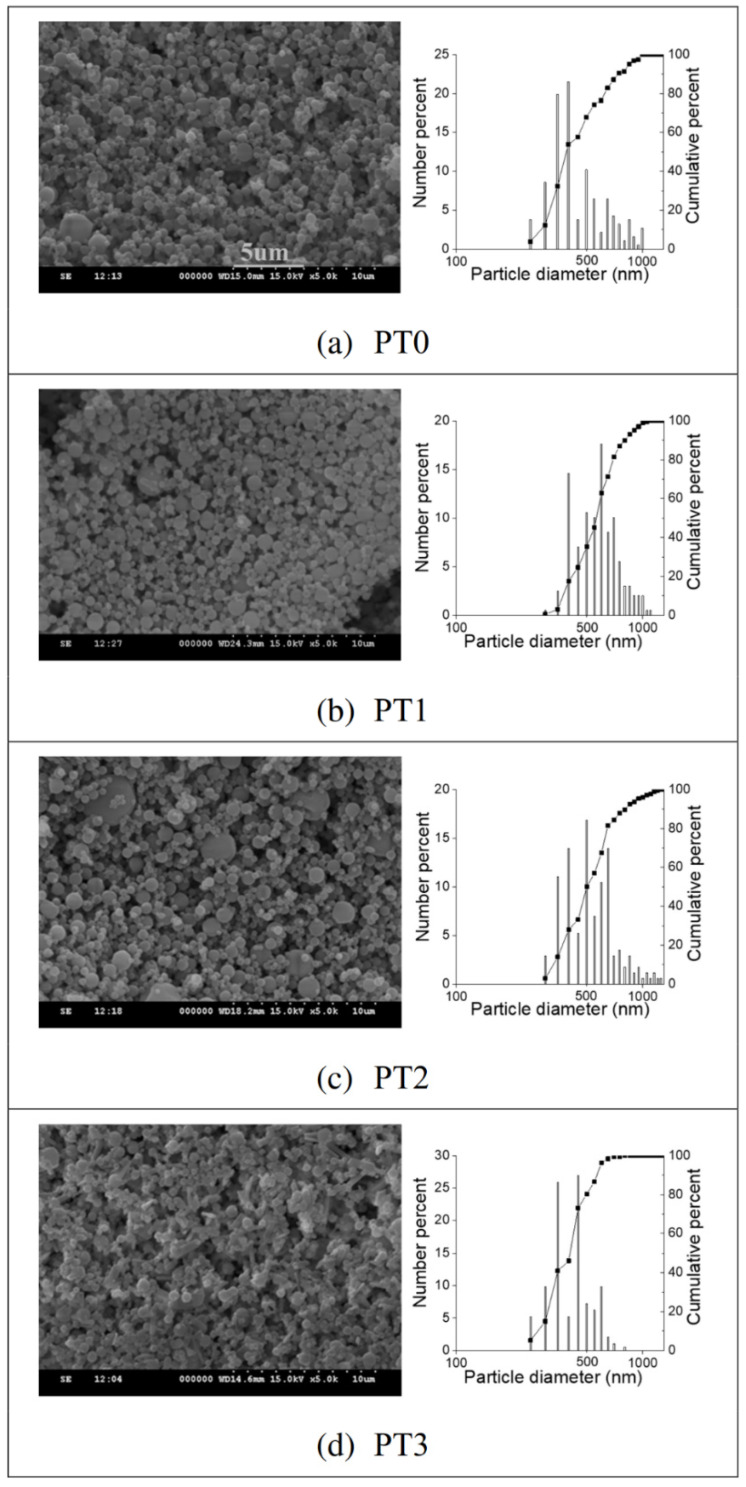
SEM images and corresponding particle size distributions of the TH-loaded pectin ((**a**): PT0), pectin/chitosan ((**b**): PT1, (**c**): PT2), and pectin/calcium ion ((**d**): PT3) submicron particles.

In this study, theophylline (TH) was selected as a model drug that was added to the pectin solution at a concentration of 0.2 mg/mL. The TH-loaded pectin particles were fabricated by adding two different crosslinking agents in another solution, namely, a high-molecular-weight cationic polysaccharide of CHC and a cationic ion of Ca^2+^ for comparison. They were prepared under the same processing conditions as mentioned above. According to the SEM observation, as shown in Figure 4, the TH-loaded pectin particles exhibited a smooth surface and spherical structure, which was similar to that without the addition of TH. When the feeding concentrations of the pectin and TH were, respectively, 0.5 and 0.2 mg/mL, the D_n_ of the pectin particles containing TH, sample PT0, was approximately 553 nm as indicated in Table 2.

If 0.1 mg/mL of CHC was further added and mixed with the solution of pectin (0.5 mg/mL) and TH, the D_n_ of the TH-loaded pectin/CHC particle (PT1) became 617 nm, and it further increased to 648 nm at a higher feeding concentration of CHC of 0.2 mg/mL (PT2) owing to the additional amount of CHC added into the particles. On the other hand, the particle size of the TH-loaded pectin particles crosslinked by calcium chloride (PT3) was smaller than that of PT1 at the same weight concentrations in the feeds. This is because CHC is a high-molecular-weight polymer that could entangle with pectin as compared to small calcium ions. Still, all the G.S.D. values were smaller than 1.5 for the TH-loaded pectin particles with a crosslinker (PT1, PT2, and PT3). It indicates that the TH-loaded pectin submicron particles with the crosslinker having a narrow particle size distribution can also be synthesized by the continuous-feeding spray-drying system with the ultrasonic atomizer.

The SEM images could provide direct information on the particle size, particle size distribution, and morphology of the TH-loaded pectin particles yet in a dry condition. The sizes of the prepared TH-loaded particles in their wet state (PBS, pH 6.8) were determined by the DLS method at 37 °C. It was found that the initial hydrodynamic diameters (HDs) of the TH-loaded pectin particles in the PBS were from approximately 630 to 712 nm, which were a little larger than those measured by the SEM images in their dry state yet with the same trend in the variation of particle size.

**Figure 5:**
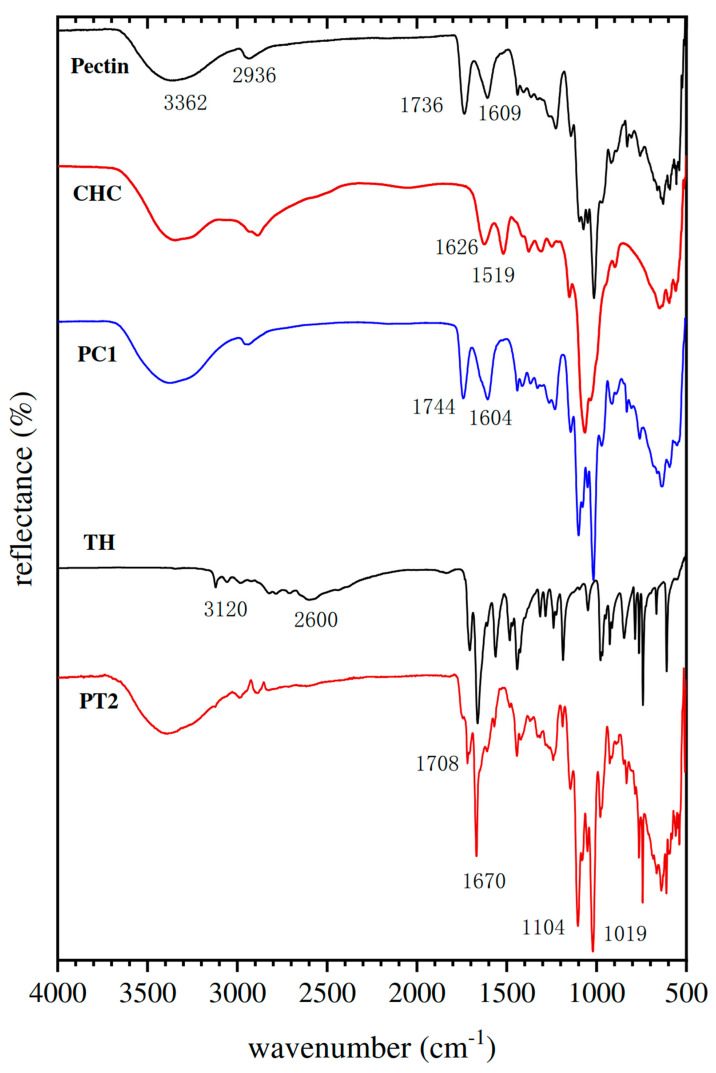
ATR−FTIR spectra of the pectin, CHC, TH particles, and samples PC1 and PT2, in which the numbers indicate the wavenumber of the peaks.

The zeta potential of the TH-loaded pectin particles was approximately −16.3 mV due to the anionic characteristic of pectin as shown in Table 2. It was neutralized by the addition of either the cationic polysaccharide of CHC or the cationic ion of Ca^2+^. It implied that the complex structures of the pectin with the cationic crosslinkers would be formed during the spray-drying process [6,8]. However, the undesired agglomeration phenomena could occur at a higher content of crosslinker in the particles. The spray-dried particles were further analyzed by using ATR-FTIR spectroscopy. As indicated in Figure 5, the spectrum of the pectin sample showed the typical undissociated carboxyl group at 1736 cm^−1^, dissociated carboxyl group at 1609 cm^−1^, and a broad peak at 3362 cm^−1^ denoting the stretching vibrations of the -OH group. For the CHC, the peak at 1626 cm^−1^ was the C=O stretching vibration of amide I, and 1519 cm^−1^ was the was the peak for -NH_3_^+^ [25]. Because the spectrum of the sample PC1 containing the pectin and CHC at the weight ratio of 5:1 indicated the change in the peak at 1519 cm^−1^, and the neutralization results observed by the zeta potential tests, it implied that the pectin/CHC complex particles could be formed by the electrostatic interaction between positively charged CHC and negatively charged pectin.

The TH-loaded pectin/CHC submicron particles were further observed using ATR-FTIR spectroscopy. The absorption bands at 1708 and 1670 cm^−1^ which referred to the carbonyl groups on the TH compound [23], and absorption peaks at 1104 and 1019 cm^−1^, corresponding to C-O groups of the CHC and pectin, were found in the IR spectrum of the TH-loaded pectin/CHC particles: sample PT2, of which the weight ratio of pectin, CHC and TH were 5:1:1. It denoted that the TH could be loaded successfully in the pectin/CHC submicron particles. The loading capacity (LC, %) is the amount of drug loaded per unit weight of the TH-loaded particles, and the encapsulation efficiency (EE, %) denotes the percentage of TH that is successfully entrapped into the polymer carriers, which can be determined according to Equations (1) and (2). As shown in Table 2, the TH loading content and efficiency were approximately 19–23% and 82–92%, respectively.

### 3.3. Release of TH from the Pectin Particles in PBS

**Figure 6:**
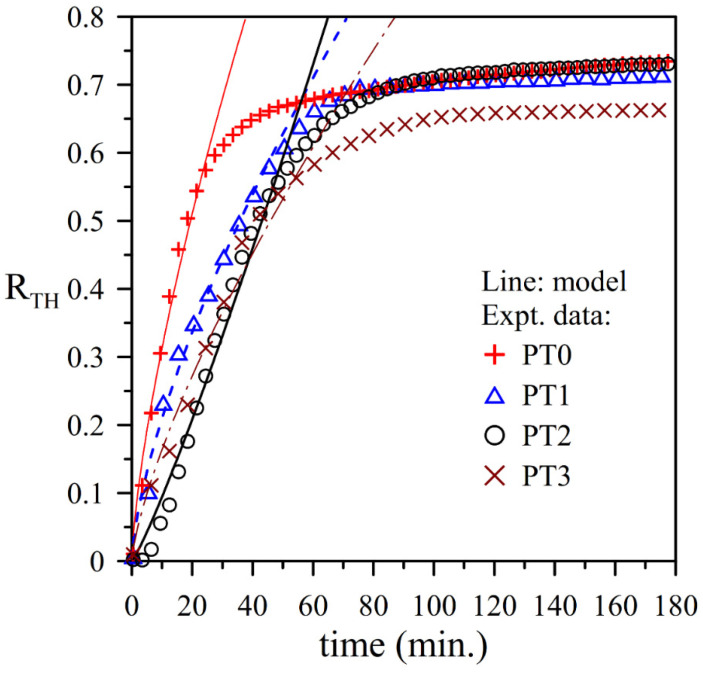
TH Fraction released from pectin, pectin/CHC and pectin/calcium ion submicron particles at various times in PBS at pH 6.8 and 37 °C.

For real-time monitoring of the TH released from the pectin/CHC submicron particles, the release profile was determined by measuring the light absorbance of the released medium at the wavelength of 272 nm using a transmission probe immersed in the PBS solution which was coupled to the UV light source. Figure 6 shows the fractional release (*R_TH_*) profiles of TH from the submicron particles with various compositions at pH 6.8 and 37 °C, in which

(3) $$R_{TH}=\frac{M(t)}{M_{\infty }}$$

and *M(t)* and $M_{\infty }$ are the masses of TH released at time *t* and the time approaches infinity, respectively.

For the TH-loaded pectin particles without any crosslinking agent (sample PT0), it was found that at the release time of 30 min, the fractional TH release was 0.61 as indicated in Table 3. With the addition of 0.1 mg/mL CHC solution in the feeding solution, the releasing rate of the TH was retarded for the sample PT1, of which the *R_TH_* became 0.44 at 30 min. It was further decreased to 0.36 when the concentration of CHC solution increased to 0.2 mg/mL (PT2). The more the crosslinking structure was formed owing to a higher concentration of CHC, the TH-loaded pectin/CHC complex particles became less flexible. In addition to the cross-linkages formed by the electrostatic interaction between the pectin and CHC, there also existed the possibility of H-bonding among the groups, such as carboxylic acid, hydroxy, methyl ester, amide, amine, carbonyl group, and imidazolium groups, within the complex of pectin, CHC and the TH drug. Therefore, the biopolymer chains formed a diffusion barrier, and the diffusion coefficient of TH decreased.

The TH-loaded pectin particles could be crosslinked with calcium ions, which resulted from the specific interaction between the calcium ions and galacturonic acid in pectin [26,27,28]. When a calcium chloride solution of 0.1 mg/mL was added to the precursor solution, sample PT3, the TH release rate became slower than that without the crosslinking agent. After 30 min, the *R_TH_* was approximately 0.38, even slower than that from the chitosan-crosslinked pectin particles at the same added amount (PT1). This indicates that the calcium-ion-crosslinked pectin particles were more compact than the chitosan-crosslinked pectin particles, restricting the diffusion of TH. Moreover, Figure 5 shows that the TH release rate gradually decreased to zero after *R_TH_* reached approximately 0.6. The release time at *R_TH_* = 0.6, denoted as t_0.6_, became slower by adding either CHC or calcium chloride to the pectin particles.

An exponential equation of the Ritger–Peppas model was proposed to describe the general drug release behavior from the polymeric matrix [29,30].

(4) $$R_{TH}=kt^{n}$$

where *k*, *n*, and *t* were the kinetic constant, diffusional exponent, and release time, respectively. Equation (4) is a simplified short-time approximation, and it is valid for the first 60% of the total drug release, that is *R_TH_* ≤ 0.6. The model was fitted to the experimental TH release data by using nonlinear least-square regression, and the calculated kinetic parameters are summarized in Table 3. The regression coefficient (R^2^) is a statistical measure of how well the regression predictions approximate the real measured data points. For the four TH-loaded submicron particles, all the values of R^2^ were greater than 0.98. The results showed good agreement between the model and experimental data. The kinetic constant k indicated the structural and geometric characteristics of the polymer matrix. It was found as 0.0615 for the TH-loaded pectin particles without any crosslinking agent (sample PT0), and the value became smaller by adding either CHC or calcium ions. The diffusional exponent *n* denoted the diffusion mechanism of the drug released from the particles. For non-swellable spherical particles, if the drug release behavior follows the Fickian diffusion, the *n* is 0.43. When the exponent *n* is greater than 0.43 and less than 1, the transport mechanism is anomalous; that is, it is neither Fickian type nor polymer chain relaxation. For zero-order drug delivery, the exponent *n* is one, which implies the drug release rate is independent of time.

For the samples PT0, PT1, and PT3, the values of *n* were 0.706, 0.681, and 0.732, respectively, as shown in Table 3. Therefore, the transport mechanism was not pure Fickian diffusion. It was believed that the drug diffusion was affected by the swelling of polymer particles in the PBS. For example, the initial mean hydrodynamic diameter of the sample PT0 measured by the DLS was approximately 630 nm, and it was swollen and became 915 nm after 30 min. In this study, a volume-swelling ratio (SR) was calculated by the initial hydrodynamic diameter (*DH*_0_) and the *DH* was measured at 30 min: *DH*_30min_ [31,32]:

(5) $$SR\%=\frac{DH_{30\min}^{3}}{DH_{0}^{3}}\times 100\%$$

It was found that the *SR* decreased with the addition of crosslinking agent. The SRs of the samples PT0, PT1, PT2, and PT3 were approximately 306%, 241%, 221%, and 182%, respectively. Increasing the concentration of crosslinking agent decreased the swelling ratio (PT1 vs. PT2). In addition, the calcium-ion-crosslinked pectin particles exhibited a lower swelling ratio indicating they provided higher crosslinking density than the CHC at the same weight concentration (PT1 vs. PT3). The TH-loaded sample PT2 was prepared from the pectin solution mixed with 0.2 mg/mL of chitosan hydrochloride solution, and the diffusional exponent *n* was 1.14. The TH transport mechanism was very close to the zero-order drug delivery, of which *n* = 1. Because the particles would be swollen during the release test in buffer, the surface area of the TH-loaded particles increased, and the crosslinked polymer matrix became looser. Therefore, the release rate of the TH would increase with time due to the swelling effect. If it could compensate for the decline in the diffusion rate resulting from the decreasing concentration gradient between the particle surfaces and the surrounding medium, it would be possible to keep the TH release at a constant rate.

## 4. Conclusions

A homemade spray drier coupled with two continuous-feeding streams was constructed. The pectin/chitosan hydrochloride (CHC) particles with diameters of approximately 300 to 800 nm were successfully prepared and tailored by changing the precursor concentration of the fed solution. The possibility of crosslinking between pectin and CHC was inhibited before the spray drying. The theophylline (TH)-loaded pectin particles with a crosslinking agent, such as CHC or calcium chloride, were further fabricated. The Ritger–Peppas model was proposed to describe the TH fractional release from the TH-loaded pectin/CHC submicron particles. For example, for the sample PT2, which was prepared from the mixture of pectin, TH, and CHC solutions of 0.5, 0.2, and 0.2 mg/mL, respectively, the diffusional exponent *n* was 1.14, indicating that the TH transport mechanism was very close to the zero-order drug delivery (*n* = 1). We believe that the structure of the spray-dried TH-loaded pectin/CHC is dependent on the pH of an aqueous solution. It is worth further investigating the influence of pH value on the release of TH.

## Figures and Tables

**Table 1:** Mean particle size (D_n_, D_VM_) and particle size distribution (G.S.D.) of pectin/CHC particles measured by SEM images.

Sample	Pectin (mg/mL)	CHC (mg/mL)	D_n_ (nm)	D_VM_ (nm)	G.S.D.
PC0	0.05	0.01	294 (±73)	296 (±95)	1.38
PC1	0.1	0.02	389 (±72)	384 (±127)	1.25
PC2	0.3	0.06	467 (±176)	485 (±144)	1.52
PC3	0.5	0.1	566 (±115)	537 (±176)	1.31
PC4	1	0.2	691 (±177)	645 (±191)	1.19
PC5	1.5	0.3	788 (±214)	763 (±225)	1.31

D_n_: number average particle diameter; D_VM_: volume mean diameter; G.S.D.: geometry standard deviation [24].

**Table 2:** Mean particle size (D_n_, D_VM_), particle size distribution (G.S.D.), loading content (LC), and encapsulation efficiency (EE) of the TH-loaded pectin particles (concentration of TH = 0.2 mg/mL).

Sample	Pectin (mg/mL)	CHC (mg/mL)	CaCl^2^ (mg/mL)	D_n_ (nm)	D_VM_ (nm)	G.S.D.	Zeta Potential (mV)	LC (%)	EE (%)
PT0	0.5	0	0	553 (±248)	558 (±161)	1.70	−16.3	23	82
PT1	0.5	0.1	0	617 (±166)	641 (±182)	1.33	−13.6	23	92
PT2	0.5	0.2	0	648 (±269)	633 (±174)	1.39	−8.40	19	86
PT3	0.5	0	0.1	502 (±128)	503 (±160)	1.27	−8.50	23	92

**Table 3:** Fraction theophylline release (R_TH_) and kinetic parameters of the Ritger–Peppas model for the theophylline-loaded pectin particles.

Sample	*R_TH_* (30 min)	*R_TH_* (180 min)	t_0.6_ (mins)	*k*	*n*	R^2^
PT0	0.61	0.73	28.5	0.0615	0.706	0.9804
PT1	0.44	0.71	48.5	0.0438	0.681	0.9852
PT2	0.36	0.72	55.0	0.0068	1.141	0.9933
PT3	0.38	0.66	66.5	0.0304	0.732	0.9887
